# Community-based audits of snake envenomations in a resource-challenged setting of Cameroon: case series

**DOI:** 10.1186/s13104-018-3409-3

**Published:** 2018-05-18

**Authors:** Frank-Leonel Tianyi, Valirie Ndip Agbor, Joel Noutakdie Tochie, Benjamin Momo Kadia, Armand Seraphin Nkwescheu

**Affiliations:** 1 Mayo Darle Sub-divisional Hospital, Banyo, Adamawa Region Cameroon; 2 Ibal Sub-divisional Hospital, Oku, North West Region Cameroon; 3 Faculty of Medicine and Biomedical Sciences, University of Yaoundé I, Yaoundé, Cameroon; 4 Foumbot District Hospital, Foumbot, Cameroon; 5 Grace Community Health and Development Association, Kumba, Cameroon; 6 Cameroon Society of Epidemiology-CaSE, P.O. Box 1411, Yaoundé, Cameroon; 7 Laboratory of Public Health Biotechnology and Research, Biotechnology Centre, University of Yaoundé 1, Yaoundé, Cameroon; 8 Research Foundation for Tropical Diseases and Environment-REFOTDE, Buea, Cameroon

**Keywords:** Snakebite, Deaths, Audit, Rural, Case series, AVS, Cameroon

## Abstract

**Background:**

Snakebites are a major cause of mortality and morbidity worldwide with the highest mortality burden in poor rural areas of sub-Saharan Africa. Inadequate surveillance systems result in loss of morbidity and mortality data in these settings. Although rarely reported in these resource-constraint environments, community-based audits are recognised pivotal tools which could help update existing data and indicate key public health interventions to curb snakebite-related mortality. Herein, we present two cases of snakebite-related deaths in a rural Cameroonian community.

**Case presentations:**

The first case was a 3-year-old female who presented at a primary care health centre and was later referred due to absence of antivenom serum (AVS). However, she had an early fatal outcome before getting to the referral hospital. The second case was an 80-year-old traditional healer who got bitten while attempting to kill a snake. He died before hospital presentation.

**Conclusion:**

Community-based audits help identify key intervention points to curb snakebite mortality in high-risk rural areas like ours. From our audits, we note a remarkable absence of affordable AVS in rural health facilities in Cameroon. We recommend frequent community health education sessions on preventing snakebites; continuous training modules for health personnel from high-risk areas; training traditional healers on the importance of AVS in managing cases of snakebite envenoming, and the need for timely hospital presentation; and setting up context-specific approaches to rapidly transport snakebite victims to hospitals.

## Background

Snake bites represent a major public health problem, disproportionately affecting poor rural communities [1–4]. Snakebite-related mortality has been associated with low socioeconomic indicators like poverty, while rural agricultural activities have been strongly linked with snakebite incidence, with farmers and children representing the most vulnerable groups [3].

The highest worldwide incidence of snakebites has been recorded in Asia, Latin America and sub-Saharan Africa (SSA) [4]. The snakebite-associated mortality in Latin America has reduced over the last decade due to the implementation of effective snakebite management systems, including the development of locally effective antivenom sera (AVS) [4]. Unlike Latin America, optimal snakebite management in SSA has been retarded by several obstacles, such as high costs of AVS, lack of government funding and incentive, deficient surveillance system for snakebites and poor healthcare-seeking behaviour of snake-bitten patients [1, 2, 4].

Mortality data and circumstances surrounding the death of snakebite victims are often difficult to come by in research [5]. National health reporting systems and hospital based studies grossly underestimate the actual burden of snakebite envenomings [1]. Well-designed community-based studies are invaluable in appreciating the circumstances surrounding a snakebite incident and the difficulties associated with access to quality healthcare, all cumulating in the death of the victim [6]. Consequently, community-based audits could complement hospital records in identifying and tailoring specific public health interventions to curb snakebite-related mortality [7]. In addition, community-based audits are easy to carry out, less costly and could help to actively involve the community in snakebite management [6].

A search of PUBMED with key words; “audit”, “snakebite”, “deaths”, “community-based interview” or “Cameroon”, revealed just a single article wherein a community-based audit was carried out for a snakebite-related death in Cameroon [8]. We present two community-based audits on snakebite-related deaths in the Mayo-Darlé health area, Adamawa Region, Cameroon. We sought to describe the circumstances surrounding the deaths of these victims, which permitted us to identify practical points through which we could ameliorate snakebite management and reduce snakebite-related mortality in rural Cameroon.

## Case presentation

### Case 1

A 3-year-old female from the Mbem tribe in the Adamawa Region of Cameroon, died on the 20/04/2017 at about 6:00 pm following a snakebite. The child was living with her grandparents who had left her at home with her cousins and gone to the farm. The oldest cousin was 9 years old. The children went to harvest palm kernels in a bush nearby their house when they saw an unidentified snake species. Upon seeing the snake, they ran leaving behind the little girl. They later came back and took her home, but since there was no adult around, they reported to no one. About 3 h later, the child presented an inability to stand, talk and open her eyes properly. The children then notified the neighbour who was a 25-year-old nursing mother. The lady upon arrival found the child lying inert on the floor with breathing difficulties. Her left leg was almost twice the size of the right one. She quickly applied a tourniquet on the left thigh and called the grandparents of the child, after which she set out immediately for the hospital.

On arrival to the health centre about 4 h after the snakebite incident, the child was unconscious with a Blantyre score of 1/5. Her left leg was almost twice the size of the right one and there was a weakly tied tourniquet on the left thigh. She had one episode of a generalized tonic–clonic convulsion while at the hospital.

Initial management consisted of a bolus of 20 ml/kg of Ringer’s lactate, anti-tetanus serum 750 IU subcutaneously, and dexamethasone 4 mg intramuscularly. The most qualified personnel on duty was a nurse assistant with no formal training on snakebite management and reporting. A lack of AVS at the health centre prompted referral to the nearest health facility with an available stock of AVS, which was about 4 h away, separated by un-motorable roads. The child died less than 30 min after leaving the health centre.

### Case 2

**Fig. 1:**
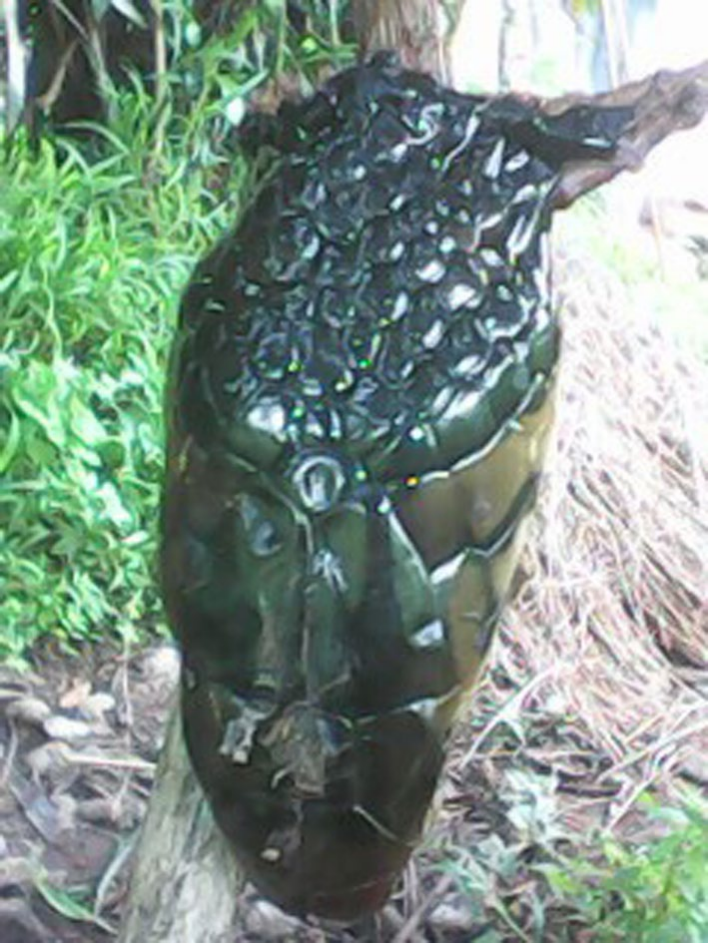
Decapitated head of snake

An 80-year-old male, who was the main traditional snakebite healer in the village died from snake envenomation on the 4/10/2016. He was shelling corn in his barn at home when he noticed an unidentified snake species. With his son, they tried to kill the snake. During the attempt, he was bitten on the left leg. They however succeeded in immobilising the snake, which they thought was dead. The victim then tried to behead the snake (probably to use as traditional remedies), and was re-bitten on his left hand. He succeeded in beheading the snake (Fig. 1) (later identified as a cobra, probably of from the *Naja melaoleuca* species). Immediate case management at home consisted of the application of traditional topical ointments on the wounds, and ingestion of oral herbal concoctions. Thirty minutes later, he complained of an inability to stand and difficulties in speaking. This was followed by a progressive decrease of consciousness. His son decided to transport him to the nearest health facility which was a primary healthcare centre, seven kilometres away. The son carried him on his back and attempted to run the distance. The victim however died about 30 min after they had left the house.

## Discussion and conclusion

We present two cases of snakebite-related deaths in rural Cameroon. The first case, a 3-year-old female who got bitten while playing with her siblings and died in the course of referral after failing to receive AVS at a health centre. The second, an 80-year-old male who got bitten while attempting to kill a snake in his home. Community-based audits were carried out to identify the circumstances surrounding the deaths of these snakebite victims.

Snakebites are a significant cause of mortality in Cameroon [9, 10]. An estimated 266 snakebite related deaths are reported annually from high-risk zones like northern Cameroon [11]. Mortality data on snakebite is scarce in Cameroon, majority of which are obtained from hospital-based sources [10]. Consequently, the values obtained usually underestimate the true burden of this neglected health problem in most communities [12–14]. This is worrisome because mortality is an important health indicator [15]. Information on the circumstance surrounding the death of snake-bitten victims could go a long way to improve on snakebite case management with a reduction in snakebite-related deaths. Rural areas account for 97% of snakebite-related deaths, however, these regions contribute little to the epidemiological picture of snakebite mortality [1]. This is because of poor health seeking behaviours, with most patients preferring traditional and herbal medicine to modern medicine, and also, many victims die before getting to the hospitals [1, 2]. These cases are unaccounted for by health statistics and underestimate the mortality burden from snakebite envenoming [1]. In the Mayo-Darle health area, a review of the monthly morbidity and mortality reports revealed no case of snakebite death in the past 5 years [16]. This was clearly not the case as evident in our report. Our series points out the need to intensify community-based research to better elucidate the burden of snakebites in Cameroon. Community-based audits permit us to better appreciate the immediate run of events leading to the death of these patients. Hence, we could plan locally adequate interventions to prevent snakebite deaths.

Children with their high rates of outdoor activities could easily encounter snakes and suffer snakebite incidents [9, 14]. Owing to their small body surface areas, they are at an increased risk of severe envenomation due to a greater amount of venom injected per unit body mass [14, 17]. In an earlier study in Cameroon, the snakebite-related mortality of children < 5 years of age was 7.1% [9, 18]. The years of life lost (YLL) following the death of a child significantly increases the burden of snakebites making snake envenoming a health priority in this population [17]. Similarly, the elderly are at an increased risk of severe outcomes following snakebites. This is due to an increased prevalence of co-morbid conditions in this population [19]. Both of our patients constituted a vulnerable population hence snakebites in these populations should be considered as a matter of urgency, for which prioritized optimal management cannot be overemphasized.

Most snakebites occur in rural areas with limited resources to manage severe cases of envenomation [1, 20]. Despite this high incidence of snakebites in rural areas, the knowledge of health personnel on its management remains inadequate. Indeed, according to a national survey conducted in 2015, a majority of Cameroonian health personnel were not versed with the latest snakebite case management options [10]. One of our victims succeeded in getting to a health centre, and was attended to by a nurse assistant who had no formal training in snakebite management. In the era of modern medicine, with the availability of safe AVS and effective ancillary treatment, it is unacceptable for a snakebite victim to be offered sub-optimal care in a health facility. The recent inclusion of snakebite envenomings in the list of neglected tropical diseases is an important step towards reducing preventable deaths from snakebite envenomings [21, 22]. This has to be accompanied by national efforts to identify gaps and provide solutions to shortcomings in snakebite management in their countries [22]. Some countries like Kenya have developed local guidelines to improve snakebite management, especially in high-risk areas [22]. This problem is being addressed by the Cameroon Society of Epidemiology which organised various training seminars for health personnel on snakebite management [16]. However, more efforts are required to reach personnel in the most remote villages, especially areas with high incidences of snakebites. Furthermore, the same victim could not benefit from AVS which was not available at the health centre. She died in the course of referral.

In the absence of affordable snake AVS in some rural areas in Cameroon [1, 12, 23], it is important to seek alternative means to decrease morbidity and mortality from snakebites [9, 10, 20]. One of such ways is to invest in community education and prevention of snakebites [13, 14, 20]. Both of our victims found themselves in compromising situations and suffered snake envenomation as a consequence. We propose a continuous training of selected health personnel. They will in-turn identify key community actors and community representatives, which they will train on the importance of preventing snakebites. In our rural poor-setting, the challenges associated with snakebite management can be tackled if the following specific interventions are put into place. Firstly, children should not be left playing unsupervised and should avoid areas where they could be at risk of snakebites such as forests, palm trees, etc. Secondly, pictures of local venomous snakes should be made available to the general population so that they can take appropriate measures to avoid snakebites when confronted with such snakes. Thirdly, high-risk actions such as beheading or attempts to kill snakes by inexperienced persons should be discouraged. Furthermore, emphasis should be made on the appropriate health-seeking behaviour following snakebites, particularly timely presentation to health facilities [1, 14, 20]. This permits rapid assessment of the severity of the snakebite envenoming and the need for snakebite AVS, thereby ensuring timely management of patients with a consequential decrease in morbidity and mortality from snakebite envenomings. In SSA, 50–90% of snakebite victims seek a traditional healer for first-line treatment [24]. Traditional healers could play a key role in improving management of snakebite victims [5]. Training them to rapidly recognize signs of severe envenoming could help reduce the delay in presentation to health facilities as they could constitute a crucial starting point for referral to health facilities. Associating alternative medicine (traditional healers) to modern medicine in snakebite case management could help reduce mortality and morbidity in poor rural settings [5]. Moreover, most snakebites often occur at locations far off from the hospital [9]. A rapid transportation system has been proven to significantly reduce mortality from snakebites [25]. Hence, it is important to get these victims to the hospital as soon as possible using appropriate means of transportation. The son of one of our victims attempted to carry his father on the back and run a distance of seven kilometres to the nearest health centre. This might have played a part in his demise. For this reason, it is crucial to put in place locally adapted algorithms or referral mechanisms to rapidly transport such victims to health facilities. A partnership between hospitals and local associations of motorbike riders to rapidly transport snakebite victims in exchange for financial motivation may be a good starting point.

In conclusion, community-based audits help identify key intervention points to curb snakebite mortality in high-risk rural areas like ours. From our audits, we note a remarkable absence of AVS in rural health facilities in Cameroon. We recommend frequent community health education sessions on preventing snakebites; continuous training modules for health personnel from high-risk areas; training traditional healers on the importance of AVS in managing cases of snakebite envenoming, and the need for timely hospital presentation; and setting up context-specific approaches to rapidly transports snakebite victims to hospitals.

## Abbreviations

SSA: sub-Saharan Africa
AVS: anti-venom serum
